# Exemplar-based judgment or direct recall: On a problematic procedure for estimating parameters in exemplar models of quantitative judgment

**DOI:** 10.3758/s13423-020-01861-1

**Published:** 2021-06-09

**Authors:** David Izydorczyk, Arndt Bröder

**Affiliations:** Department of Psychology, Experimental Psychology Lab, School of Social Sciences, University of Mannheim, D-68131 Mannheim, Germany

**Keywords:** Judgment, Exemplar model, Recall

## Abstract

Exemplar models are often used in research on multiple-cue judgments to describe the underlying process of participants’ responses. In these experiments, participants are repeatedly presented with the same exemplars (e.g., poisonous bugs) and instructed to memorize these exemplars and their corresponding criterion values (e.g., the toxicity of a bug). We propose that there are two possible outcomes when participants judge one of the already learned exemplars in some later block of the experiment. They either have memorized the exemplar and their respective criterion value and are thus able to recall the exact value, or they have not learned the exemplar and thus have to judge its criterion value, as if it was a new stimulus. We argue that psychologically, the judgments of participants in a multiple-cue judgment experiment are a mixture of these two qualitatively distinct cognitive processes: judgment and recall. However, the cognitive modeling procedure usually applied does not make any distinction between these processes and the data generated by them. We investigated potential effects of disregarding the distinction between these two processes on the parameter recovery and the model fit of one exemplar model. We present results of a simulation as well as the reanalysis of five experimental data sets showing that the current combination of experimental design and modeling procedure can bias parameter estimates, impair their validity, and negatively affect the fit and predictive performance of the model. We also present a latent-mixture extension of the original model as a possible solution to these issues.

In their everyday lives, people have to make judgments of different importance in a variety of domains and situations. For instance, customers in a restaurant have to predict how tasty a meal will be, doctors have to judge the severity of a patient’s disease, and employers have to judge how well a possible employee will perform in the future. Such judgments require inferring a continuous criterion (e.g., tastiness) from a number of cues (e.g., is there cheese on it or not) of a given judgment object.

One process people may rely on to make these judgments is based on previously encountered objects and their criterion values stored in memory (Juslin, Olsson, & Olsson, 2003; Juslin, Karlsson, & Olsson, 2008). New objects are then judged based on the similarity to these exemplars (Juslin et al., 2003). For example, a diabetic person needs to judge the amount of carbohydrates in a dish to estimate the amount of insulin she has to apply. To do so when confronted with a new meal, she might think of previous meals (i.e., the memorized exemplars) and compare them to the current meal. The amount of carbohydrates of the new meal will then be judged according to the similarity of this new meal to past meals in memory and their respective amount of carbohydrates (i.e., the criterion value of the exemplars), whereby more similar past meals will have a stronger influence on the judgment than dissimilar ones.

Such a judgment strategy is usually described by exemplar models (e.g., Juslin et al., 2003; von Helversen & Rieskamp, 2008). Exemplar models have originally been used in many different domains such as memory (e.g., Hintzman, 1984) and categorization and classification (e.g., Medin & Schaffer, 1978; Nosofsky, 1984). One exemplar model originally used for modeling stimulus categorization, the Context Model (Medin & Schaffer, 1978), has been extended to account for data of continuous judgments from multiple cues (Juslin et al., 2003; Juslin & Persson, 2002). During the last two decades, this or related exemplar models have been used to describe one possible cognitive process in studies of multiple-cue judgments as an important area in judgment and decision-making (JDM) research (e.g., Bröder & Gräf, 2018; Hoffmann, von Helversen, & Rieskamp, 2013; Juslin et al., 2003; Mata, von Helversen, Karlsson, & Cüpper, 2012; Pachur & Olsson, 2012; Persson & Rieskamp, 2009; von Helversen & Rieskamp, 2009).

In the current paper, we argue that the usual practice of how these exemplar models are used in multiple-cue judgment research in combination with the paradigm commonly used in this field of research leads to biased estimation and impaired validity of parameters. We claim that the problems we tackle here are particularly pronounced in the multiple-cue judgment literature as compared to categorization research where the model and the experimental paradigm originated (Juslin et al., 2003). We highlight a severe problem of the application of these exemplar models in JDM research in the following respects: First, we will briefly describe the typical experimental paradigm and modeling procedure used in multiple-cue judgment studies and introduce the context model (Medin & Schaffer, 1978) as an example for an exemplar-based model how it is used in this line of research (e.g., Bröder & Gräf, 2018; Juslin et al., 2003; von Helversen & Rieskamp, 2008; Wirebring, Stillesjö, Eriksson, Juslin, & Nyberg, 2018). We will then illustrate how this paradigm with the currently applied specification of the exemplar model can potentially distort parameter estimation. Second, we will present simulation results demonstrating biased estimation and impaired validity of parameters. In addition, since many multiple-cue judgment studies use model-fit indices like the RMSE to compare different judgment process models (e.g., Hoffmann, von Helversen, & Rieskamp, 2014; Mata et al., 2012; Wirebring et al., 2018) we will also look at the model fit and predictive performance of the model. Third, we will present the results of five reanalyzed experimental data sets demonstrating that this problem also threatens the interpretation of real data. As a remedy, we will present a latent-mixture extension of the original model, as a possible solution to these problems. Although the focus of this paper lies on the multiple-cue judgment literature and how the exemplar models are applied there, we will also discuss if and how the results of this work might extend to other research areas where these exemplar models are applied.

## Typical design and estimation procedure in multiple-cue judgment studies

A typical experiment in the multiple-cue judgment literature employing exemplar models consists of at least two phases: a training phase and a testing phase. In the training phase, participants have to learn the cues and the criterion values of some stimuli (i.e., the exemplars), as well as the relationship between the cues and the criterion. This is typically done by repeatedly judging the criterion values of a sample of objects and receiving trial-by-trial-feedback. Long training phases, sometimes in combination with a learning criterion or performance contingent payment, are used to ensure intensive learning and memorization of the exemplars by the participants (e.g., Bröder, Newell, & Platzer, 2010; Hoffmann et al., 2013; Wirebring et al., 2018). In the testing phase, participants then have to judge the criteria of new stimuli and of already-learned exemplars. For instance, in Study 2 of von Helversen and Rieskamp (2009), participants had to evaluate the quality of job candidates (i.e., the criterion) on a scale of 1 to 100. The fictitious job candidates varied on six different cues (e.g., knowledge of programming languages, C++ vs. Java, knowledge of foreign languages, French vs. Turkish, etc.). The training phase consisted of 20 blocks with eight trials each. In each trial, participants had to judge one of the eight job candidates (i.e., the exemplars). After each trial, the participants received feedback about the number of points this candidate should receive and how close their estimate had been. In the testing phase, participants then had to judge 30 job candidates twice. From these 30 candidates, 22 were new candidates and eight were exemplar candidates from the training phase.

The parameters of the model of interest are often estimated based on the data of the last training blocks (e.g., Hoffmann et al., 2013; Juslin et al., 2008). These estimated parameters are then used to predict the data of each participant in the testing phase to avoid overfitting. The goodness-of-fit is then determined, for instance, via the root-mean-squared error (RMSE) between the model prediction and the participants’ actual judgments or the Bayesian Information Criterion (BIC, Schwartz, 1978). The goodness-of-fit criteria are then often used together with qualitative indices of extrapolation and interpolation (e.g., Bröder & Gräf, 2018; Juslin et al., 2003) to compare the exemplar model with other possible judgment-process models, such as a rule-based model (e.g., Juslin et al., 2003; Hoffmann et al., 2013). Qualitative indices of extrapolation and interpolation are a valuable addition to quantitative goodness-of-fit measure, since exemplar models cannot predict judgments for new objects that extend beyond the range of learned criterion values, whereas rule-based models can. Hence, testing for extrapolation in human judgments can help to distinguish the processes.

## Exemplar model used in multiple-cue judgment research

The exemplar model we use as an example in this paper is based on the context model of Medin and Schaffer (1978) extended to account for the continuous criterion in multiple-cue judgments (Juslin et al., 2003). This and similar exemplar models have been used in many studies of multiple-cue judgments, where it is assumed that judgments are based on the memory of previously encountered exemplars (e.g., Bröder & Gräf, 2018; Bröder, Gräf, & Kieslich, 2017; Juslin et al., 2003; Hoffmann et al., 2013; Hoffmann et al., 2014; Hoffmann, von Helversen, Weilbächer, & Rieskamp, 2018; Karlsson et al., 2008; Platzer & Bröder, 2013; von Helversen & Rieskamp, 2008; von Helversen, Mata, & Olsson, 2010; Wirebring et al., 2018). According to this model, when a judgment is made about a probe (i.e., a stimulus that has to be judged), the judge considers the similarity of the probe to all of the previously encountered exemplars. Similarity then acts as a weight on the stored criterion values. When applied to a continuous criterion in a multiple-cue judgment task, the stored criterion value of a similar exemplar in memory has more influence on the judged criterion value of the probe, whereas the criterion value of a dissimilar exemplar receives less weight (Juslin et al., 2003). The similarity between a probe and an exemplar is determined by feature overlap. An exemplar with large feature overlap is more similar to the probe and thus has more impact on the judgment.

Regarding the formal definition, the model is based on the similarity *S* between a probe and the exemplars. It is assumed that the probe serves as a retrieval cue, activating previously encountered exemplars in memory. The probe $\vec {p}$ and each exemplar $\vec {e_{j}}$ are represented by vectors of *D* binary cues ∈{0,1}. The similarity parameters *s*_*i*_, *i* = 1,...,*D*, are the only free parameters in this model, defined on the interval [0,1]. They determine how strongly a mismatch of objects on cue *i* influences the similarity *S* that can vary between 0 and 1. For simplicity, we assume the *s*_*i*_ to be constant across cues, that is, *s*_*i*_ = *s*, for all *s*_*i*_ (e.g., Bröder & Gräf, 2018; Juslin & Persson, 2002; von Helversen & Rieskamp, 2008).^1^ The similarity $S(\vec {p},\vec {e})$ between $\vec {p}$ and one exemplar $\vec {e}_{j}$ is determined according to the similarity rule of the context model (Medin & Schaffer, 1978):

1 $$ S(\vec{p},\vec{e_{j}}) = \prod\limits_{i=1}^{D} d_{i} \text{ with } d_{i} = \begin{cases} 1 \text{ if } p_{i} = e_{i} \\ s \text{ if } p_{i} \neq e_{i} \end{cases} $$

where *D* is the number of cues of each object. For binary cues this simplifies to:

2 $$ S(\vec{p},\vec{e_{j}}) = s^{D-m} $$

where *m* is the number of matching cues between $\vec {p}$ and $\vec {e}_{j}$. The judged criterion value $c^{\prime }$ of the probe $\vec {p}$ is then the average of all *n* exemplar criterion values $\vec {c}$ in memory, weighted by the similarity of the respective exemplar to the probe:

3 $$ c^{\prime} = \frac{{\sum}_{j=1}^{n} S(\vec{p},\vec{e_{j}})*c(\vec{e_{j}})}{{\sum}_{j=1}^{n} S(\vec{p},\vec{e_{j}})} $$

where $c(\vec {e_{j}})$ is the criterion value of exemplar *j*. Equation 3 is the extension of the context model (Medin & Schaffer, 1978) from binary to a continuous criterion as suggest by Juslin et al. (2003; see also Elliot & Anderson, 1995; Juslin & Persson, 2002). It involves many simplifying assumptions, such as not directly modeling the exemplar retrieval process (cf., the EBRW model of Nosofsky & Palmeri, 1997), assuming that all exemplars are used when making a judgment (cf., Nosofsky & Palmeri, 1997; Albrecht, Hoffmann, Pleskac, Rieskamp, & von Helversen, 2019), and that all exemplars, their cues, and their criterion values are remembered and recalled without error. However, a detailed modeling of the recall and retrieval process is not intended with this model as it is used in the multiple-cue judgment literature, since it is mainly used as a tool to classify rule- and exemplar-based processes of judgments.

### The *s* parameter

The *s* parameter from the model above is often called similarity parameter, since from an analytical point of view, it controls the similarity between two exemplars (Medin & Schaffer, 1978, see the example below). Psychologically, the *s* parameter has been interpreted as an attention parameter, since the perceived similarity of two exemplars decreases, when more attention is paid to potential cue mismatches (Medin & Schaffer, 1978; see also Juslin et al., 2003; von Helversen & Rieskamp, 2009). However, the *s* parameter can also be seen as a continuous measure of memory discriminability, where high values indicate no discrimination between exemplars and very small values indicate a perfect discrimination between exemplars in memory. The memory discriminability of exemplars increases when exemplars become well-learned as their memory traces become more distinct, reducing the perceived similarity (Shiffrin, Clark, & Ratcliff, 1990; Kılıç, Criss, Malmberg, & Shiffrin, 2017). The results reported in this article are relevant regardless of the preferred interpretation of *s* as either a memory or as an attention parameter.

**Fig. 1:**
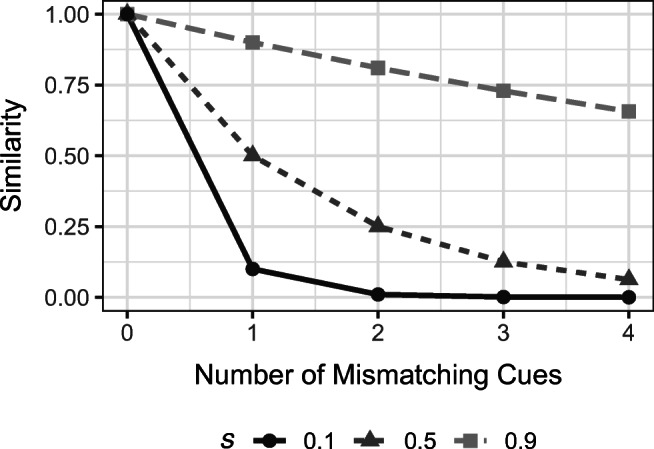
The similarity between two stimuli for different numbers of mismatching cues and different values of *s* according to the context model (Medin & Schaffer, 1978)

To illustrate, Fig. 1 depicts the similarity between two hypothetical stimuli $\vec {a}$ and $\vec {b}$ with four cues each, for different numbers of mismatching cues (i.e., 0, 1, 2, 3, or 4), plotted for different values of *s*. For *s* = .9 (i.e., low discriminability), the similarity decreases rather slowly. However, for *s* = .1 (i.e., high discriminability), even a mismatch of only one cue (e.g., $\vec {a}$ = [1,1,1,1] and $\vec {b}$ = [0,1,1,1])) leads to a large decrease in similarity from 1 to 0.1. As more and more cues mismatch, the similarity asymptotically approaches 0. Due to the multiplicative combination of the mismatches (see Eq. 1), smaller values of *s* lead to a much steeper decrease of similarity with each mismatch and hence, much less influence of dissimilar exemplars on the judgment of the probe. This also implies that if *s* is equal, or very close to 0, only perfectly matching or very similar exemplars (if existent) will determine the judgment, otherwise, judgments are erratic. If *s* is equal, or very close to 1, every exemplar has the same influence, thus resulting in the prediction of the mean of the exemplar criterion values for each and every probe.

## The psychological misspecification of the exemplar model in multiple-cue judgment research

The exemplar model as described above assumes that judging the criterion value of a probe *always* involves the reconstruction of the criterion value as a similarity-weighted average of all stored exemplar criterion values. This, however, seems psychologically implausible in typical multiple-cue judgment tasks where participants are repeatedly confronted with the same small set of judgment objects during the training phase. In this situation, we think that it is more realistic to assume that more and more exemplars become well learned, and when a probe is presented which is identical to one of the overlearned exemplars in memory, the criterion value of this very exemplar will be retrieved rather than building a similarity-weighted average of all exemplars. Hence, we assume that depending on the strength of an exemplar’s memory representation, one of two qualitatively distinct processes will take place: If a strongly represented exemplar is found in memory which exactly matches the probe, the participant will simply recall this single exemplar’s criterion value and report it as their judgment. We will henceforth refer to this process as *“(direct) recall”*. In the alternative case if there is no strong representation of a perfectly matching exemplar (the exemplar has not been overlearned, yet, or the probe is new), the similarity-weighted reconstruction as described in the original exemplar model takes place. Hence, all exemplars are recalled (cf., Nosofsky & Palmeri, 1997; Albrecht et al., 2019), and the judgment is built buy the similarity-weighted averaging process, for ease of differentiation, we will henceforth simply call this second process *“judgment”*. Of course, this judgment is also based on the recall of the exemplars. But it entails the additional process of a similarity-weighted integration of their criterion values in contrast to the “direct recall”-process mentioned above, which only entails the retrieval of just this one specific well-learned exemplar and then reproducing its retrieved criterion value. For ease of presentation, we use the shortcut terms “judgment” for the former integration process and “direct recall” for the latter simple recall process involving only the identical exemplar. Our conjecture is that lumping these qualitatively different processes together into one may severely distort the characterization of the process as well as the estimate of the similarity parameter *s*.

The reason for this conjecture is that the exemplar model can account for both types of processes (judgment and direct recall). In the judgment process, the response is a similarity-weighted average based on all exemplars. In the direct-recall process, it is only the identical exemplar that governs the response. Which of those two modes of aggregation is used depends on the *s* parameter and how the proposed psychological misspecification then might affect the estimation of the *s* parameter can be demonstrated with an example. Assume there are two exemplars and one to-be-judged stimulus with two binary cues as shown in Table 1. The to-be-judged stimulus is identical to Exemplar 1. Based on Eqs. 2 and 3, we can generate predictions for the unknown criterion value of the to-be-judged stimulus for a very high and a very low value of *s*. For *s* = 1 we get a prediction of $c^{\prime } = 5$, which is the mean of the criterion values of the two exemplars. For *s* = 0 we get $c^{\prime } = 3$, which is the criterion value of Exemplar 1. This implies that a very small *s* value leads to the prediction of the exact criterion value of the matching exemplar (if existent). Thus, when estimating the parameters from data by minimizing the distance between observed data and model-implied predictions, which can be seen as the reverse of prediction, *s* has to be as small as possible if two conditions are met: A probe is identical to one of the exemplars and the judged criterion of the probe is equal to the true criterion value of the matching exemplar. One instance where these conditions apply is when a participant has learned an exemplar and its respective criterion in an earlier stage of the experiment, and then later, when presented with the same exemplar again, recalls the learned criterion.

**Table 1:** Cue and criterion values for two exemplars and one probe

	Cue 1	Cue 2	Criterion
Exemplar 1	0	1	3
Exemplar 2	1	1	7
Probe	0	1	**?**

Note. The probe is identical to the first exemplar

Therefore, we conjecture that the estimation of the *s* parameter is biased towards 0 if the responses of participants include direct recall of exemplars and their criterion values, and if all data points are jointly used to estimate the parameter. Furthermore, we predict that this problem is aggravated with increasing numbers of recalled exemplars, since there are more cases influencing the estimation of the *s* parameter. In addition, the model should show decreased model fit and make less accurate predictions when based on these biased parameter estimates.

In the present work, we show that disregarding the distinction between similarity-based judgment and direct recall leads to large errors in the estimation and impaired validity of model parameters. For this, we first present results from a computer simulation testing these predictions, showing bias in parameter estimation and how to avoid it. Next, we reanalyze data from five experiments and show the differences in parameter estimation and model fit between the usual procedure and a redefined procedure.^2^

## Simulation

In this section, we show the severity of the problem and address the adequacy of a solution by running a computer simulation. The goal was to measure the bias in the estimation of the *s* parameter, for different true values of *s* and different recall probabilities *P*_*r*_ (i.e., the probability that the criterion value of an exemplar is recalled correctly). We compared three different ways for estimating the *s* parameter of the exemplar model presented above.

First, we used the typical procedure of multiple-cue judgment studies described above, which estimates the model parameters of the original exemplar model based on all data points regardless if it was a directly recalled exemplar, a not recalled exemplar, or a new stimulus. Based on the reasoning presented before, we expected that, when this $\hat {s}_{orig}$ parameter is estimated in this usual manner, it is more biased towards 0, the more correctly recalled exemplars there are in the data.

Second, since we propose that correctly recalled exemplars lead to a biased estimation of the *s* parameter, as a simple proof-of-concept, we estimated two different *s* parameters by splitting the data into two distinct sets of stimuli: Recalled exemplars (i.e., the well-learned, very distinct exemplars) versus not recalled exemplars and the new stimuli (i.e., less well-learned and less discriminable exemplars, as well as new stimuli). The $\hat {s}_{split}$ parameter, estimated only on the data set with not recalled exemplars and the new stimuli, should then be an unbiased estimator of *s*. However, this simple proof-of-concept is based on post hoc evaluation of the data (i.e., the classification in correctly recalled exemplar and other) and also reduces the amount of data used for estimating the parameter, since only a subset of the data is used. This method of splitting the data can be used as a heuristic remedy to arrive at appropriate estimates of *s*, however, if the extended model described next cannot be applied.

**Fig. 2:**
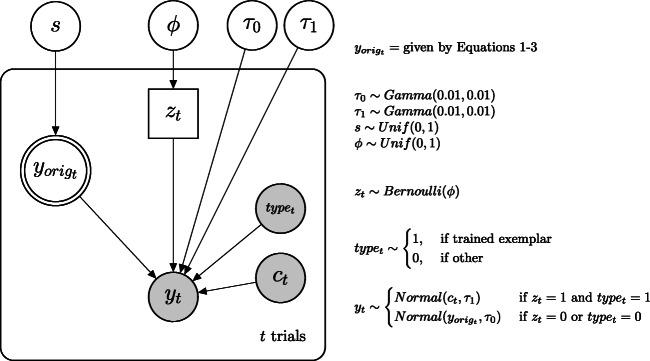
Graphical model of the latent-mixture extension of the original exemplar model

As a more elegant solution, we also used an extended version of the exemplar model which directly integrates the assumption that there are two distinct processes at work when people are confronted with already presented stimuli.^3^ The graphical model is depicted in Fig. 2. This latent-mixture model (Zeigenfuse & Lee, 2010) assumes that the final response *y*_*t*_ of a participant in a trial *t* is generated by one of two possible processes, if the stimulus in this trial was part of the training phase: A direct retrieval of the learned criterion value of this trained exemplar *c*_*t*_ (= direct recall) or the similarity-weighted reconstruction as described in the original exemplar model $y_{orig_{t}}$ (= judgment). Which data generating process is used, given the stimulus in this trial was a trained exemplar, is determined by an indicator variable *z*_*t*_. If *z*_*t*_ = 0 the data *y*_*t*_ follow a normal distribution with precision *τ*_0_ and centered around the prediction of the original exemplar model $y_{orig_{t}}$, which is based on the parameter *s*. If *z*_*t*_ = 1 the data *y*_*t*_ follow a normal distribution with precision *τ*_1_ and centered around the learned criterion value of this exemplar *c*_*t*_. The indicator *z*_*t*_ follows a Bernoulli distribution with parameter *ϕ*. This parameter *ϕ* represents the latent memory probability; this is the probability that a trained exemplar is directly recalled and the corresponding criterion value reproduced. To summarize, this extended latent-mixture model of the original exemplar model integrates the assumption that if a probe in a trial is a novel stimulus, the similarity-weighted average response based on the original exemplar model is used based on the parameter *s*. When the probe is a trained exemplar, the response is the directly recalled learned criterion value of this exemplar with probability *ϕ* and the similarity-weighted average response based on the original exemplar model with probability 1 − *ϕ*. The estimated $\hat {s}_{int}$ parameter should then also be unbiased, since the possibility of direct retrieval is already integrated into the model.

### Procedure

In this simulation, we generated behavioral data by manipulating two independent variables (the true value of *s* and the probability that the criterion value of an exemplar is recalled correctly) and investigated how these variables influence the parameter estimation. A summary of the simulation procedure is shown in Algorithm 1 in the Appendix. In the first step of this simulation, we generated the stimulus matrix, consisting of 32 stimuli that can be created with five binary cues. The criterion values were computed according to a linear additive rule:

4 $$ \begin{array}{@{}rcl@{}} c &=& w_{0} + cue_{1} \times w_{1} + cue_{2} \times w_{2} + cue_{3} \times w_{3} + cue_{4}\\ && \times w_{4} + cue_{5} \times w_{5}. \end{array} $$

where *cue*_*i*_ represents the binary cues and *w*_*i*_ the corresponding cue weights. Of the 32 stimuli, 12 are randomly selected as to-be-learned exemplars. In order to create realistic stimulus material used in actual experiments (e.g., Bröder et al., 2017; Bröder & Gräf, 2018), the four most extreme stimuli (i.e., the two stimuli with the highest and the two stimuli with the lowest criterion value) were never selected as exemplars. In addition, there was also a switch of criterion values between one pair of stimuli (i.e., if one stimulus *a* of this switch pair would have a criterion value of 31 and stimulus *b* of the pair a value of 59 , the new values after switching would be 59 for *a* and 31 for *b*). The cue weights *w*_*i*_ for cues *i* = 0,...,5 had to sum to 100 and were randomly drawn from a truncated normal distribution with *μ* = 20, *σ* = 10, an upper bound of 100, and a lower bound of 0.

In the second step, we generated judgment data from this stimulus matrix according to Juslin et al.’s. (2003) version of the context model (Medin & Schaffer, 1978) presented above. The true *s* parameter varied in 4 steps from a very strict similarity criterion to a more lenient criterion, *s* = .001, .01, .3 or .8. The recall probability (*P*_*r*_) could either be .1, .5 or 1. This means that, for instance for *P*_*r*_ = 0.5, there is a probability of .5 that an exemplar and its corresponding criterion value is recalled correctly and, therefore, there could be more or less than 50% of correctly recalled exemplars in a given iteration of the simulation when *P*_*r*_ = .5. A value of *Pr* = 1 indicated that every exemplar (and its criterion value) is recalled correctly and the judged criterion value of this exemplar is therefore its exact criterion value. A value of *Pr* = .1 indicates that only very few exemplars are recalled exactly.^4^ It should be noted that a recall probability of 1 is what most studies aim for when applying an extensive training phase. Also, as most exemplar models are based on the assumption that all exemplars and their corresponding criterion values are remembered correctly and are all used in the subsequent judgment process (cf., Nosofsky & Palmeri, 1997; Albrecht et al., 2019), participants should learn all exemplars correctly. Note also that we added no additional error to the generated judgment data, so in principle one would expect perfect parameter recovery.

In the third step, we estimated the $\hat {s}$ parameters with JAGS (Plummer, 2003) interfaced with R using the *runjags* package (Denwood, 2016), using each of the three methods. The results are based on MCMC chains with 5000 samples from each of two independent chains collected after 5000 burn-in samples were discarded, 5000 adaptive iterations, and thinning by recording every 5th sample. The convergence of the chains was checked by visual inspection and the standard $\hat {R}$ statistic (Brooks & Gelman, 1998).

In the final step, we computed the Bayes factors for model comparison between the original exemplar model (${\mathscr{M}}_{0}$) and the latent-mixture model (${\mathscr{M}}_{1}$). Since the original exemplar model is nested within the latent-mixture model when *ϕ* = 0, we computed the Bayes factor based on the Savage–Dickey density ratio (Wagenmakers, Lodewyckx, Kuriyal, & Grasman, 2010; Vandekerckhove et al., 2015):

5 $$ BF_{10} = \frac{p(\phi=0|\mathcal{M}_{1})}{p(\phi=0|D,\mathcal{M}_{1})} $$

where $p(\phi =0|{\mathscr{M}}_{1})$ is the density of the prior distribution of *ϕ* at 0 given ${\mathscr{M}}_{1}$, $p(\phi =0|D,{\mathscr{M}}_{1})$ is the density of the posterior distribution of *ϕ* at 0 given ${\mathscr{M}}_{1}$, and *BF*_10_ is the Bayes factor in favor of ${\mathscr{M}}_{1}$. The density of the posterior distribution was computed with the *dlogspline* function in the *polspline* package in R (Kooperberg, 2020). Since we used a uniform (0,1)-prior for *ϕ*, the density $p(\phi =x|{\mathscr{M}}_{1})$ on any given point *x* is 1. The resulting Bayes factor BF_10_ then indicates the evidence of ${\mathscr{M}}_{1}$ compared to ${\mathscr{M}}_{0}$, or how much more probable the data are under ${\mathscr{M}}_{1}$ compared to ${\mathscr{M}}_{0}$ (Kass & Raftery, 1995; Morey, Romeijn, & Rouder, 2016; Vandekerckhove et al., 2015). For instance, a Bayes factor of BF_10_= 10 would indicate that the data are 10 times more likely to occur under ${\mathscr{M}}_{1}$ than under ${\mathscr{M}}_{0}$. In addition, we computed the root-mean-squared-error (RMSE) between the actual data and the median of the posterior predictive distribution in each trial of both models as an indicator for the prediction error margin of each model and since the RMSE is often used in multiple-cue judgment studies for model comparison (e.g., Hoffmann et al., 2013; Wirebring et al., 2018; von Helversen & Rieskamp, 2009).

All steps were repeated 200 times for each combination of true *s* parameter and *P*_*r*_ value, which leads to 200 × 4 × 3 = 2400 simulated data sets in total. For each simulated data set, a new stimulus matrix, with different exemplars, cue weights, and criterion values was generated as described in the first step. The code of the simulation, the JAGS model codes, example of MCMC chains and $\hat {R}$ values of all three estimation methods for a randomly selected iteration of the simulation, and the results are available at the Open Science Framework (https://osf.io/b69f3/).

### Results

#### Recovery *s*

**Fig. 3:**
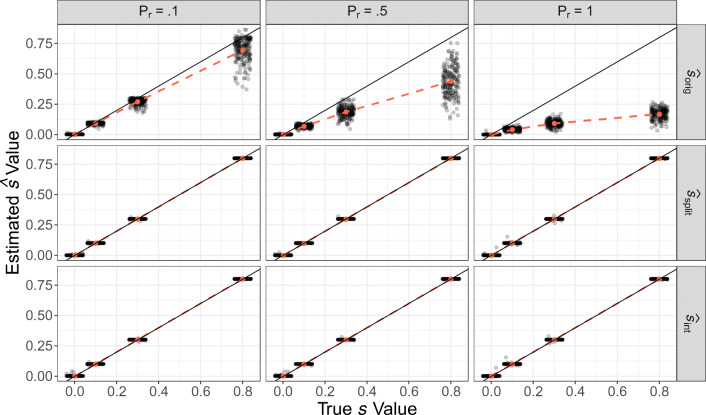
Estimated *s* values for different true *s* values and different *P*_*r*_ for three different types of *s* parameter. The *black solid lines* represent what would be expected for perfect parameter recovery. *Red points* and *dashed lines* show and connect the means of 200 repetitions. $\hat {s}_{orig}$ is the estimated *s* parameter based on the original exemplar model. $\hat {s}_{split}$ is the estimated *s* parameter based on the original exemplar model, when only the subset of the data without any recalled exemplars was used. $\hat {s}_{int}$ is the estimated *s* parameter of the latent-mixture extension of the original exemplar model

The results of the simulation are displayed in Fig. 3 and Table 2. The first row in Fig. 3 shows that the recovered parameter $\hat {s}_{orig}$ of the original exemplar model was very close to the true *s* values, when *P*_*r*_ was small. However, with an increasing percentage of correctly recalled exemplars (*P*_*r*_), $\hat {s}_{orig}$ increasingly deviated from *s*, with larger deviations for larger *s* values. For a high recall probability of *P*_*r*_ = 1, $\hat {s}_{orig}$ deviated strongly from the true *s* and was severely biased downward towards 0. In addition, $\hat {s}_{orig}$ was never larger than .17 for *s* = .3 and .27 for *s* = .8 when *P*_*r*_ = 1, which is less than half as large as the actual true value. Thus, the first row in Fig. 3 shows that the estimated $\hat {s}_{orig}$ parameter is a severely biased estimator of *s* if judgment and direct recall (or the data generated by these processes) are mixed. The bias of $\hat {s}_{orig}$ increases, when more exemplars are recalled directly. Yet, a good memory performance is exactly the goal researchers try to achieve, when they design their experiments with an extensive learning phase.

**Table 2:** Means (and standard deviations) of the estimated *s* values for different true *s* values and different memory probabilities

		true *s*			
*P*_*r*_	type	.001	.1	.3	.8
	$\hat {s}_{orig}$	.001 (.002)	.092 (.008)	.272 (.026)	.696 (.096)
0.1	$\hat {s}_{split}$	.001 (.002)	.100 (.001)	.300 (.002)	.800 (.000)
	$\hat {s}_{int}$	.001 (.004)	.100 (.002)	.300 (.002)	.800 (.000)
	$\hat {s}_{orig}$	.001 (.001)	.069 (.012)	.186 (.045)	.439 (.121)
0.5	$\hat {s}_{split}$	.001 (.002)	.100 (.001)	.300 (.002)	.800 (.000)
	$\hat {s}_{int}$	.001 (.004)	.100 (.001)	.300 (.002)	.800 (.000)
	$\hat {s}_{orig}$	.001 (.002)	.042 (.013)	.093 (.027)	.169 (.044)
1.0	$\hat {s}_{split}$	.002 (.005)	.100 (.004)	.300 (.003)	.800 (.001)
	$\hat {s}_{int}$	.001 (.005)	.100 (.004)	.300 (.003)	.800 (.001)

Note. *P*_*r*_ indicates the recall probability. Type indicates the type of *s* parameter: $\hat {s}_{orig}$ is the estimated *s* parameter based on the original exemplar model. $\hat {s}_{split}$ is the estimated *s* parameter based on the original exemplar model, when only the subset of the data without any recalled exemplars was used. $\hat {s}_{int}$ is the estimated *s* parameter of the latent-mixture extension of the original exemplar model

The second row of Fig. 3 shows the estimated $\hat {s}_{split}$ parameter, the parameter that was estimated only on the subset of the data without any directly recalled exemplars. When the parameter is estimated based only on new stimuli and criterion values that are not perfectly recalled, the recovered $\hat {s}_{split}$ values were identical to the true values, see again Table 2 for the descriptive values. Although not shown in Fig. 3, the estimated $\hat {s}_{split}$ parameter values based on the subset of only correctly recalled exemplars were mostly estimated close to 0, as to be expected.

The third row of Fig. 3 shows the recovered $\hat {s}_{int}$ parameter values from the latent-mixture extension of the original exemplar model. The results in Fig. 3 and displayed in Table 2 show that the true *s* parameter values are recovered very well by $\hat {s}_{int}$ when the possibility of direct recall of trained exemplar was integrated into the model. In addition, we found that the *ϕ* parameter was very close to *P*_*r*_ on average over all iterations, except for *s* = .001, where the average *ϕ* was below .1, regardless of *P*_*r*_ (see Table S1 in the online supplementary material). We assume this is since the difference in criterion values between a directly recalled exemplar and the predicted value based on the exemplar model with *s* = .001 can be rather small (e.g., 47 and 47.002, see Table S2 in the online supplement) and the model then tends to classify directly recalled exemplars as “not-recalled” (i.e., *z*_*t*_ = 0).

#### Model comparison

The Bayes factors as a mean of model comparison between the original exemplar model and the latent-mixture extension are shown in Table 3. When the data-generating *s* parameter was very small, the Bayes factor favors on average the original exemplar model, indicated by the negative *log*(BF_10_), regardless of *P*_*r*_. This is because the *s* parameter is with .001 already very close to 0, thus there is not much room for the downward biasing effect of correctly remembered exemplars and the Bayes factors then favors the less complex original exemplar model with fewer parameters. However, the average *log*(BF_10_) increasingly favors the latent-mixture model with an increasing number of correctly recalled criterion values, with values more or less being constant for the different possible true *s* values.

**Table 3:** Means (and standard deviations) of the *log*(*BF*_10_)

	true *s*			
*P*_*r*_	.001	.1	.3	.8
0.1	− 2.32 (3.11)	2.92 (2.13)	3.06 (1.86)	3.13 (1.82)
0.5	− 1.83 (4.29)	8.74 (1.94)	8.76 (1.91)	8.71 (1.79)
1.0	− 2.30 (3.03)	18.45 (1.6)	18.55 (1.55)	18.49 (0.68)

Note. *P*_*r*_ indicates the recall probability

We can find a similar pattern for the RMSE shown in Table 4. When the data were generated with a very small *s* value, we get a similar average low RMSE for the original exemplar model and its latent-mixture extension. However, the RMSE of the original exemplar model increases the larger the *s* parameter and the number of correctly recalled exemplars become.

**Table 4:** Means (and standard deviations) of the RMSE of the original exemplar model (orig) and the latent-mixture extension with integrated direct recall process (int)

		true *s*			
*P*_*r*_	Type	.001	.1	.3	.8
0.1	$RMSE_{{\mathscr{M}}_{0}}$	0.03 (0.17)	0.70 (0.44)	1.67 (0.92)	3.36 (1.7)
	$RMSE_{{\mathscr{M}}_{1}}$	0.03 (0.16)	0.04 (0.25)	0.02 (0.14)	0 (0.01)
0.5	$RMSE_{{\mathscr{M}}_{0}}$	0.08 (0.35)	1.25 (0.33)	3.17 (0.75)	6.06 (1.29)
	$RMSE_{{\mathscr{M}}_{1}}$	0.08 (0.35)	0.02 (0.14)	0.01 (0.06)	0 (0.01)
1.0	$RMSE_{{\mathscr{M}}_{0}}$	0.04 (0.22)	1.40 (0.36)	3.23 (0.68)	6.13 (1.17)
	$RMSE_{{\mathscr{M}}_{1}}$	0.04 (0.21)	0.04 (0.23)	0.02 (0.11)	0 (0.01)

Note. *P*_*r*_ indicates the recall probability. ${\mathscr{M}}_{0}$ is the original exemplar model and ${\mathscr{M}}_{1}$ the latent-mixture extension

### Discussion

We ran a simulation to investigate the potential bias in the estimation of the *s* parameter, when one does not differentiate between recalled exemplars and not recalled exemplars as well as new stimuli.

The results suggest that the estimation of the *s* parameter as well as predictions based on this estimation can be inaccurate, when the distinction between directly recalled exemplars and judgment is not taken into account. The deviation of $\hat {s}_{orig}$ from *s* was small when either the true *s* parameter was small, or when *P*_*r*_ was small, that is, when there were only very few directly recalled exemplars. However, we found large biases in estimation as well as in predictions when there was a medium to large recall probability and true *s* value. The results show that the estimated $\hat {s}_{orig}$ is biased downwards, when the true *s* parameter and the recall probability was large. However, this large recall probability, as stated before, is exactly the outcome many experimenters aim for when designing their experiments with extensive learning phases: Many studies implement a learning criterion that participants have to reach to advance to the next phase of the experiment or terminate the learning phase before the maximal number of learning blocks (e.g., Bröder et al., 2010; Hoffmann et al., 2013; Wirebring et al., 2018). In addition, the number of learning blocks (i.e., the number of times an exemplar is presented) can range from 4 (Pachur & Olsson, 2012) up to 40 blocks (Wirebring, Stillesjö, Eriksson, Juslin, & Nyberg, 2018), with most studies using 8–10 blocks. Participants are instructed, and with these extensive learning phases also able, to memorize these stimuli and their respective criterion values. For instance, in Experiment 1B in Bröder, Gräf, and Kieslich (2017) participants showed an average correct recall rate (what we called here the recall probability *P*_*r*_) of .79 (*SD* = .23) and 46.67% had a correct recall rate of .90 or more. Furthermore, it is not the recall probability per se, but the relative number of correct exemplars to all trials, that is, how many data points from all possible trials are correctly recalled exemplars, which drives this effect. The more recalled exemplars there are in the data, the stronger $\hat {s}_{orig}$ is biased towards 0. For instance, in our simulation, with 32 stimuli, 12 exemplars, and *P*_*r*_ = 1, there were $\frac {12}{32} = 37.50$% correctly remembered exemplars. The fact that parameters are often estimated on the data of the training blocks (e.g., Bröder & Gräf, 2018; Juslin et al., 2003; von Helversen & Rieskamp, 2008), where *all* stimuli are exemplars, makes this finding even more alarming. These findings could in principle explain why many studies find small $\hat {s}_{orig}$ values, since based on the results of the simulation, small $\hat {s}_{orig}$ values can arise both, from small true *s* values, but also from larger true *s* values, when combined with the often-achieved high number of correctly recalled exemplars in the data.

The results show that this bias in $\hat {s}_{orig}$ is caused by correctly remembered exemplars, this is instances where the judgment of the criterion value of a trained exemplar is identical to its true criterion value, since the bias disappears when the $\hat {s}_{split}$ was estimated only on the subset of the data without any recalled exemplars. As a more elegant solution, using the here presented latent-mixture extension of the original exemplar model where the possibility of correctly recalling a trained exemplar is integrated into the model also lead to an unbiased estimation of the *s* parameter. In fact, the few deviations of the estimated $\hat {s}_{int}$ from the underlying *s* parameter are probably due to the random simulation procedure and instances of unfortunate selections of exemplars and combinations of generated criterion values, which would not be used in a real experimental setting.

In the next section, we investigate if these effects reported here are likely to be found in real experimental data as well, by reanalyzing existing data from five different multiple-cue judgment experiments.

## Re-analysis

We reanalyzed data from five different experiments from Bröder et al., (2017), Bröder and Gräf (2018), and one unpublished data set from the same lab group. The aim was to investigate if the effects found in the simulation extend to empirical data as well. Before we describe our general approach, we first outline the experimental procedure used in one of the experiments. The material and procedure in the other experiments were very similar to the one described below and can be found in the corresponding papers, or, for the unpublished data set, in the supplemental material. The code and results are again available at the Open Science Framework (https://osf.io/b69f3/).

### Materials and procedure of the reanalyzed data sets

In Experiment 1A of Bröder et al., (2017), participants had to judge stimuli on a scale from 0 to 100 based on a set of four binary symptoms (e.g., fever vs. hypothermia), resulting in 16 different stimuli. They either had to judge the severity of a patient’s disease or the toxicity of a bug. Since cue patterns and criterion values of both stimulus sets were identical and for reasons of simplicity, we will not make a distinction between the content domain in the subsequent analysis. The experiment itself consisted of three phases: a training phase, a decision phase, and a final testing phase. In the training phase, participants had to judge severity of illness of eight patients or the toxicity of eight bugs (the exemplars) and feedback about the actual criterion value was provided. Participants were instructed to either use the feedback about the correct criterion values to learn a mathematical rule connecting cue and criterion values (rule condition) or to memorize the objects and their values (exemplar condition). The training phase consisted of eight blocks with eight trials each (one for each exemplar). In the decision phase, participants had to choose the stimulus with the higher criterion value of 45 pairs of objects. These data, however, are not important for the current project. In the testing phase, participants had to judge the criterion values of all 16 stimuli (i.e., exemplars as well as new stimuli). They were instructed to either apply the mathematical rule they learned earlier (rule condition) or judge untrained objects by their similarity to the memorized objects (exemplar condition).

### Method

Because of the often-documented “rule-bias” and since we were interested in the exemplar model, we chose the conditions of the five experiments in which exemplar-based processing was expected (or shown) to be most prevalent. We selected the data from the corresponding exemplar condition from each experiment, where participants were either directly instructed to use an exemplar-based approach (e.g., Experiment 1A in Bröder et al., 2017), or where an exemplar-based strategy was induced by experimental design (e.g., Bröder and Gräf, 2018). For instance, in Bröder and Gräf (2018), we only used the data from the condition where a dimensional cue format was combined with memory-based judgments, as more exemplar-based reasoning has been observed under these conditions (Bröder, Newell, & Platzer, 2010; Platzer & Bröder, 2013). See Table 5 for a short overview of all experiments and the selected conditions.

**Table 5:** Sample size, mean (and standard deviation) of proportion of correctly recalled exemplars, names and short description of the selected condition of each experiment

Exp.	Label	*n*	*P*_*r*_	Selected condition	Short description of condition
Bröder et al., (2017) - 1A	171A	62	.43 (.32)	Exemplar instruction	Participants were instructed to use an exemplar-based strategy.
Bröder et al., (2017) - 1B	171B	30	.85 (.15)	Exemplar instruction	Participants were instructed to use an exemplar-based strategy.
Bröder et al., (2017) - 2	172	30	.69 (.31)	With picture	Each exemplar was always accompanied by a picture of a male person to facilitate exemplar-based processing.
Bröder and Gräf (2018)	18	30	.78 (.28)	Memory-based dimensions	A dimensional cue format was combined with memory-based judgments, to facilitate more exemplar-based reasoning.
Bröder and Gräf (unpublished)	XX	35	.54 (.30)	Long learning phase	Participants had a longer training phase to facilitate exemplar storage and thus exemplar-based processing.

Note. Label indicates the respective abbreviations used in subsequent tables and figures

*n* represents the respective sample size. *P*_*r*_ represents the proportion of correctly recalled exemplars

We then again used JAGS (Plummer, 2003) to fit the original exemplar model and the latent-mixture model depicted in Fig. 2 to the data of the judgment phase of each experiment. We ran two MCMC chains with 5000 samples each with thinning by recording every 5th sample, after 15,000 burn-in samples and 15,000 adaptive iterations. The convergence of the chains was checked by visual inspection and the standard $\hat {R}$ statistic (Brooks & Gelman, 1998). We also computed Bayes factors for model comparison between the original exemplar model (${\mathscr{M}}_{0}$) and the latent-mixture model (${\mathscr{M}}_{1}$), using the Savage–Dickey density ratio (Wagenmakers et al., 2010). In addition, we computed the RMSE between the actual data and the median of the posterior predictive distribution of each model.

### Hypotheses

We had three predictions based on the simulation results reported before. First, regarding the *s* parameter, we expected to find higher values for $\hat {s}_{int}$ than for $\hat {s}_{orig}$, since $\hat {s}_{orig}$ should be biased towards 0 when there are correctly recalled exemplars in the data (H1). Second, since $\hat {s}_{orig}$ becomes smaller on average when there are more correctly recalled exemplars, but $\hat {s}_{int}$ does not depend on the number of correctly recalled exemplars (see Fig. 3), we expected to find a negative correlation between $\hat {s}_{orig}$ and the number of correctly recalled exemplars. We also expected to find no such correlation with $\hat {s}_{int}$ and the number of correctly recalled exemplars (H2). Third, we expected to find that the data are better predicted by the latent-mixture model than by the original exemplar model, as indicated by a positive *log*(BF_10_) (H3).

### Results

#### H1. Differences in *s* Parameter

**Fig. 4:**
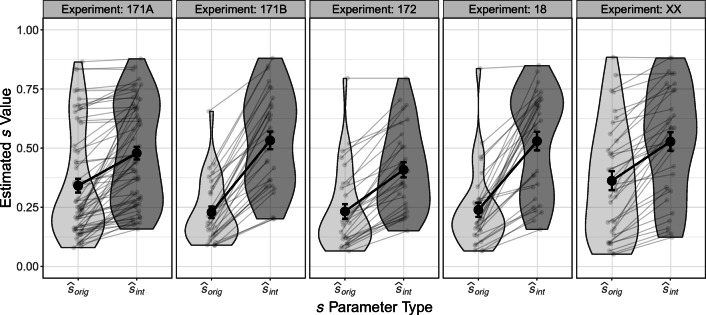
Median posterior values of $\hat {s}_{int}$ and $\hat {s}_{orig}$ for each participant and for each data set. Black dots represent the means and the corresponding standard errors

We conducted one-sided paired-samples *t*-tests for the differences between $\hat {s}_{int}$ and $\hat {s}_{orig}$ for each experiment. The results, together with the descriptive values, are shown in Table 6. Overall, we found significant differences between $\hat {s}_{int}$ and $\hat {s}_{orig}$, with all *p* s < .001 and *d* s ≥ 1.02. The median posterior estimates of $\hat {s}_{int}$ and $\hat {s}_{orig}$ for each person and each experiment depicted in Fig. 4 show that as hypothesized $\hat {s}_{int}$ is larger than $\hat {s}_{orig}$ for almost all participants. Indeed, there is only one instance were $\hat {s}_{int}$ (0.881) was smaller than $\hat {s}_{orig}$ (0.884) and this is for a participant who did not recall any exemplar correctly (i.e., *P*_*r*_ = 0). In addition, there was a very high correlation between the estimated latent memory probability parameter *ϕ* of the latent-mixture model and the empirical proportion of correctly recalled exemplars of each participant (Table 6), which supports the validity of the *ϕ* parameter.

**Table 6:** Means (and standard deviations) for different *s* parameters, test statistics and effect sizes of the difference between $\hat {s}_{int}$ and $\hat {s}_{orig}$ for five data sets. Means (and standard deviations) of the latent memory parameter *ϕ* and its correlation with the empirical proportion of correctly recalled exemplars

					*s* parameters					
Exp.	*n*	*P*_*r*_	*ϕ*	$r_{P_{r}\times \phi }$	$\hat {s}_{orig}$	$\hat {s}_{int}$	*t*	*df*	*p*	*d*
171A	62	.43 (.32)	.45 (.28)	.93 [.88,.96]	.34 (.23)	.48 (.21)	8.00	61	<.001	1.02
171B	30	.85 (.15)	.79 (.13)	1.00 [1.00,1.00]	.23 (.13)	.53 (.20)	12.88	29	<.001	2.35
172	30	.69 (.31)	.66 (.26)	1.00 [1.00,1.00]	.23 (.17)	.41 (.18)	10.79	29	<.001	1.97
18	30	.78 (.28)	.74 (.24)	1.00 [1.00,1.00]	.24 (.16)	.53 (.22)	9.36	29	<.001	1.71
XX	35	.54 (.30)	.53 (.25)	1.00 [1.00,1.00]	.36 (.24)	.53 (.23)	8.33	34	<.001	1.41

Note. *n* represents the respective sample size. *P*_*r*_ represents the proportion of correctly recalled exemplars

#### H2. Correlations

**Fig. 5:**
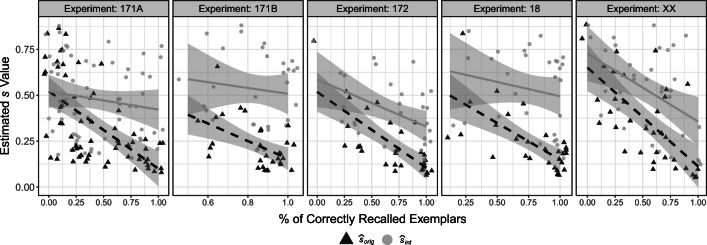
Median posterior values of $\hat {s}_{int}$ (grey dots) and $\hat {s}_{orig}$ (black triangles) by proportion of correctly recalled exemplars, for each participant and for each data set. Lines and shaded areas represent the simple linear regression estimate and the 95% confidence interval

To test our additional predictions we calculated the correlation between $\hat {s}_{int}$ as well as $\hat {s}_{orig}$ and the number of correctly recalled exemplars across participants and then compared these two correlations with the test proposed by Dunn and Clark for the difference between two overlapping correlations based on dependent groups (Dunn & Clark, 1969; Hittner, May, & Silver, 2003). The results are shown in Table 7. In every data set, we found a stronger negative correlation of $\hat {s}_{orig}$ (range: -.55 to -.75) with the number of correctly recalled exemplars than for $\hat {s}_{int}$ (range: -.12 to -.47), with differences ranging from .20 to .48, *ps* ≤ .001. Furthermore, as evident from Fig. 5, we found a similar pattern as in the simulation where there seems to be an upper bound for $\hat {s}_{orig}$ for high numbers of correctly recalled exemplars (see Fig. 3). Also evident from Fig. 5 is that, other than expected, there were two instances where $\hat {s}_{int}$ was still significantly related to the number of recalled exemplars.

**Table 7:** Correlations [and 95*%* CI] of $\hat {s}_{int}$ and $\hat {s}_{orig}$ with the number of correctly recalled exemplars and the test statistics regarding their differences in each data set

			*r*				
Exp.	*n*	*P*_*r*_	$\hat {s}_{orig}$	$\hat {s}_{int}$	Δ	*t*	*p*
171A	62	.43 (.32)	−.60 [−.74,−.41]	−.15 [−.39,.10]	.45	6.65	<.001
171B	30	.85 (.15)	−.55 [−.76,−.23]	−.12 [−.46,.25]	.43	3.88	<.001
172	30	.69 (.31)	−.75 [−.87,−.53]	−.45 [−.70,−.10]	.30	4.46	<.001
18	30	.78 (.28)	−.68 [−.83,−.42]	−.20 [−.52,.17]	.48	3.58	<.001
XX	35	.54 (.30)	−.68 [−.82,−.44]	−.47 [−.70,−.17]	.20	2.99	.001

Note. *n* represents the respective sample size. *P*_*r*_ represents the proportion of correctly recalled exemplars. Δ represents the difference between the correlations

However, as these are only correlational findings, the data from the unpublished experiment allowed us to address this prediction (i.e., that the number of correctly recalled exemplars affects $\hat {s}_{orig}$ but not $\hat {s}_{int}$) experimentally. This experiment consisted of two conditions which only differed in the length of the training phase (4 vs. 8 blocks, see the supplemental material for a more detailed description). This difference in length of the training phase should lead to a lower number of correctly learned exemplars for participants with a shorter training phase. This difference in the number of correctly learned exemplars should then lead to lower $\hat {s}_{orig}$ values when participants had a longer training phase and thus recalled more exemplars correctly, but it should not affect $\hat {s}_{int}$. As expected, participants with a shorter training phase recalled fewer exemplars in the final testing phase correctly (*M* = 2.29,*SD* = 1.82) than participants with a longer training phase (*M* = 4.29,*SD* = 2.38), *t*(63.66) = − 3.94, *p* < .001, *d* = 0.94.

In addition, consistent with the previous results, the difference between $\hat {s}_{orig}$ and $\hat {s}_{int}$ was larger for participants with eight training blocks than for participants with only four training blocks (*F*(1,68) = 7.72, *MSE* = 0.01, *p* = .007, $\hat {\eta }^{2}_{G} = .006$), as $\hat {s}_{orig}$ was lower in the long training condition (*M* = 0.36,*SD* = 0.24) than in the short training condition (*M* = 0.45,*SD* = 0.24), but there was no difference for $\hat {s}_{int}$ (*M*_*short*_ = 0.54,*SD* = 0.23, *M*_*long*_ = 0.53,*SD* = 0.23).

#### H3. Model comparison

**Fig. 6:**
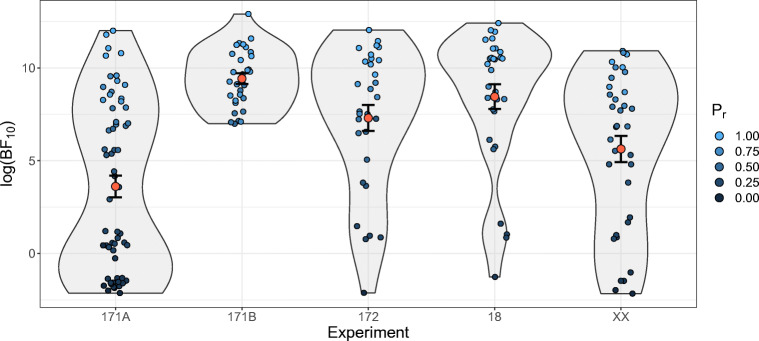
The *log*(BF_10_) colored by the proportion of correctly recalled exemplars (*P*_*r*_) for each participant and for each data set. The *red dots* represent the means and the corresponding standard errors

As expected, the latent-mixture model was on average better able to account for the data, as indicated by the high positive *log*(BF_10_), ranging from $M_{log(\text {BF}_{{10}})} = 3.61$ in Experiment 171A to in Experiment 171B $M_{log(\text {BF}_{{10}})} = 9.42$, see Table 8 for the full results. As evident from Fig. 6, there is some variation in the extent to which the latent-mixture model is better able to account for the data of individual participants, with even some instances where the original exemplar model was better able to predict their data. However, Fig. 6 does also show that these differences are mostly due to the difference in the proportion of correctly recalled exemplars of the participants ($r_{log(\text {BF}_{{10}}) \times P_{r}} = .97$, *t*(185) = 52.80, *p* < .001). Furthermore, we found significant differences between the RMSE of the both models, with the latent-mixture model having a lower RMSE on average, *p* s ≤ .002, *d* s ≥ .56.. However, as evident from Table 8, although we found the expected differences in all data sets, some differences were rather small, for instance in Experiment 2 of Bröder et al., (2017).

**Table 8:** Means (and standard deviations) of the RMSE of the original exemplar model or the latent-mixture model with integrated recall, with the corresponding test statistics and effect sizes of the difference between them, as well as the *log*(BF_10_), for five data sets

			*log*(BF_10_)				*RMSE*					
Exp.	*n*	*P*_*r*_	*M*	*SD*	*Min*	*Max*	${\mathscr{M}}_{0}$	${\mathscr{M}}_{1}$	*t*	*df*	*p*	*d*
171A	62	.43 (.32)	3.61	4.59	− 2.14	12.01	13.88 (3.61)	13.25 (3.59)	7.07	61	<.001	0.90
171B	30	.85 (.15)	9.42	1.58	6.99	12.91	11.62 (3.45)	10.40 (3.32)	5.51	29	<.001	1.01
172	30	.69 (.31)	7.30	3.84	− 2.12	12.04	12.56 (3.17)	12.19 (3.02)	3.08	29	.002	0.56
18	30	.78 (.28)	8.45	3.65	− 1.27	12.41	12.27 (3.30)	11.20 (3.23)	5.53	29	<.001	1.01
XX	35	.54 (.30)	5.63	4.20	− 2.17	10.92	13.73 (3.86)	12.86 (3.54)	4.99	34	<.001	0.84

Note. *n* represents the respective sample size. *P*_*r*_ represents the proportion of correctly recalled exemplars. ${\mathscr{M}}_{0}$ is the original exemplar model and ${\mathscr{M}}_{1}$ the latent-mixture extension

### Discussion

We reanalyzed data from five different experiments to investigate if the effects found in the previous simulation extend to empirical data as well. Results showed large differences between $\hat {s}_{orig}$ and $\hat {s}_{int}$ in all five data sets: $\hat {s}_{orig}$ was estimated to be smaller than $\hat {s}_{int}$ in each data set, as was suggested by the simulation and theoretical reasoning. It is also notable that the higher the proportion of correctly recalled exemplars was, the larger was the difference between $\hat {s}_{orig}$ and $\hat {s}_{int}$. Furthermore, correlational results on the participant level also showed that $\hat {s}_{orig}$ highly depends on the number of correctly recalled exemplars, with participants who recalled more exemplars correctly having lower $\hat {s}_{orig}$ values. Although not always independent from the number of recalled exemplars as originally expected, $\hat {s}_{int}$ was clearly less strongly correlated with the number of recalled exemplars. These results were further corroborated with experimental data of one data set showing that participants with a longer training phase recalled more exemplars correctly and had lower $\hat {s}_{orig}$ values than participants with a shorter training phase. Yet, there was no difference in $\hat {s}_{int}$. One might argue that it is plausible that participants who better learned the exemplars are better able to differentiate between them, which in turn is captured by lower *s* values in the model. Although this might be true, we still would argue that the simulation results presented before clearly show that the relationship between a higher number of correctly recalled exemplars and lower $\hat {s}_{orig}$ values can be a pure methodological and technical artifact. Taken together, these results would also suggest that the difference between $\hat {s}_{orig}$ and $\hat {s}_{int}$ would be even larger when parameters are estimated on data from the training phase (by $\hat {s}_{orig}$ becoming even smaller), since there are only trained exemplars in the learning phase and thus, the bias of $\hat {s}_{orig}$ can be even greater.

Moreover, we found that overall and for most individual participants, the latent-mixture model integrating a direct recall process of trained exemplars is better able to account for the data, where for participants with a very low number of correctly recalled exemplars the original exemplar model was preferred. However, although the Bayes factors give strong to extreme (overall) evidence for the latent-mixture model, the differences in RMSE of both models, which is often used in multiple cue judgment studies as a goodness-of-fit criterion, were rather small for some experiments, although we found somewhat larger differences in the simulation.

To further investigate this, we ran a simulation similar to the one described before, but with settings based on the experiments we reanalyzed. That is, we used the same stimuli, exemplars, and criterion values as in the studies we reanalyzed. In addition, in each of the 500 repetitions of the simulation we drew the recall probability *P*_*r*_ and the true *s* value from *beta* distributions with similar means and standard deviations as found in the experiments we reanalyzed^5^. We then used these parameters and the stimuli to generate judgment data in each repetition according to the exemplar model presented in Eqs. 1 to 3 and then added normal distributed error with *μ* = 0 and $\sigma \sim N(17,6)$^6^ with a lower bound of 0 and upper bound of 100. To be clear, we only defined the stimuli, the criterion values, *s*, and *P*_*r*_, based on the data of the reanalyzed experiments. However, we did not define or set any constraints on the resulting RMSE. We then estimated the parameters and assessed the RMSE as in the simulation reported above. The results are shown in Table 9. As intended, the average *s* parameters over all simulations were similar to the average values found in the empirical experiments. Furthermore, we found that although the overall RMSE was a little bit higher in the simulation, the average effect sizes of the RMSE difference between both models and the average *log*(BF_10_) were similar to the ones found in the empirical data sets. This suggests that the results we found regarding the differences in RMSE and *log*(BF_10_) are somewhat typical for the specific memory performance observed in the studies and the specific stimuli and range of criterion values used in the experiments we reanalyzed.

**Table 9:** Means (and standard deviations) of parameter estimates, RMSE, and effect sizes, from simulated as well as empirical data

	*s* parameters		RMSE			
Type	$\hat {s}_{orig}$	$\hat {s}_{int}$	${\mathscr{M}}_{0}$	${\mathscr{M}}_{1}$	*d*	*log*(BF_10_)
Empirical	.28 (.19)	.50 (.21)	12.81 (3.48)	11.98 (3.34)	0.86 (0.86)	6.88 (3.57)
Simulation	.32 (.19)	.52 (.20)	13.72 (4.62)	12.76 (4.82)	1.13 (0.27)	6.42 (3.45)

Note. *d* represents Cohen’s *d* for a paired-sample *t* test. ${\mathscr{M}}_{0}$ is the original exemplar model and ${\mathscr{M}}_{1}$ the latent-mixture extension

A limitation of the results presented here is that the procedure of the experiments we reanalyzed were very similar to one another. Also, despite having different content domains, the stimuli used in all experiments (i.e., number of cues, number of stimuli, exemplars, and criterion values) were the same in all experiments. Therefore, it is still open to which extent the results generalize to other experiments, with different stimuli, cues, and exemplars.

## General discussion

We proposed that in the typical experimental procedure in the multiple-cue judgment literature, the responses of participants are a mixture of two qualitatively distinct cognitive processes (similarity-based judgments and direct recall) and that disregarding this distinction can lead to biased estimation and impaired validity of parameters. We ran a simulation and reanalyzed data from five experiments to investigate the properties and extents of this issue, as well as the adequacy of a solution. Results of the simulation and the reanalysis showed that the estimation of the *s* parameter of the context model (Medin and Schaffer, 1978) extended to account for the continuous criterion in multiple-cue judgments (Juslin et al., 2003) can be severely biased towards 0 and that the model fit decreases if one does not differentiate between recalled exemplars and other stimuli, especially for larger values of the underlying *s* parameter and if more exemplars are recalled correctly. Furthermore, we found that on an individual level, the usually estimated $\hat {s}_{orig}$ parameter was very strongly negatively correlated with the number of correctly recalled exemplars in all five data sets, whereas the redefined parameter $\hat {s}_{int}$ showed a weaker to no relationship. The simulation and the reanalyzed data sets showed that the predictive performance of the exemplar model is impaired when one does not differentiate between recalled exemplars and other stimuli.

These findings have several implications. First, we showed that the standard procedure for estimating the *s* parameter can lead to biased parameter estimates and impaired fit of the model. However, this is not a problem with the model itself. The problem is rather the adaptation of the experimental design from categorization research which involves having few overlearned stimuli (e.g., Medin & Schaffer, 1978; Nosofsky & Palmeri, 1998), to multiple-cue judgment research, in order to apply the exemplar models also to multiple-cue judgments (Juslin et al., 2003). The important difference between categorization and judgment is the scale of the criterion. In categorization research the criterion is categorical, for instance, two categories A or B (e.g., Medin & Schaffer, 1978; Juslin et al., 2003; Smith & Minda, 1998). In this case, multiple exemplars share the same criterion value, since there are several exemplars in category A and several exemplars in category B. Thus, there is no unique exemplar-criterion-value combination as in the multiple-cue judgment literature, were most exemplars have their unique criterion value. This combination of very few well learned exemplars with their unique criterion values leads to the biased estimation of the *s* parameter we presented here. We would thus propose that the bias of the *s* parameter is less profound in a categorization experiment, where multiple exemplars share the same criterion. In addition, there are also other paradigms were the bias of the *s* parameter should be not necessarily a problem. For instance, if participants get no direct feedback about the criterion value, they are not able to just learn and recall the exact criterion value (e.g., Pachur & Olsson, 2012). Also, there are studies in the multiple-cue judgment and in the categorization literature, were stimuli are often defined by continuous dimensions such as length, size, and brightness rather than by binary features (e.g., Brehmer, 1972; Nosofsky & Alfonso-Reese, 1999; Ratcliff & Rouder, 1998), which leads to a large set of unique stimuli and exemplars, which also makes it harder for participants to learn specific exemplars and their criterion values. However, on a psychological level, the mixture between different process still is a problem in these cases.

Second, the findings presented in this work could explain why previous studies found rather small values for the *s* parameter of the exemplar model. For instance, von Helversen and Rieskamp (2008) found average estimated parameter values between .001 and .17 (according to von Helversen & Rieskamp, 2009), Juslin, Karlsson, and Olsson (2008) found average values from .14 to .36, and Bröder and Gräf (2018) found an average *s* value of .11. As evident from Fig. 3, when participants recalled most of the exemplars the estimated parameter becomes rather small, even when the true underlying *s* value was large. In the simulation, there was an upper bound of .27 for the estimated $\hat {s}_{orig}$ parameter when *s* = .8 and when the recall of exemplars was perfect.

Third, because of the biased estimation of the model parameter, the goodness-of-fit and predictive performance of the model are impaired. But having non-biased parameter estimates becomes important since indices of model fit and model comparison (e.g., RMSE, BIC, BF) are often used to classify participants as users of a rule-based or an exemplar-based process (e.g., Hoffmann et al., 2013; von Helversen & Rieskamp, 2008; Wirebring et al., 2018). For example, von Helversen and Rieskamp (2008) estimated the parameters of different candidate models (e.g., the exemplar model introduced here) by minimizing the RMSE for participants’ judgments in the last three blocks of the training phase. They then compared the RMSE between the model predictions and the actual data in the test phase to determine which process participants relied on. The predictions for the test phase were based on the estimated parameters of the training phase. By neglecting the different retrieval-based processes and estimating only one distorted *s* parameter, the exemplar model may suffer an undeserved disadvantage in the model comparisons which in the end could even result in an overestimation of rule-based processes in judgment. However, this problem may be less severe in studies comparing rule-based and exemplar-based models by qualitative indices of extrapolation and interpolation (e.g., Bröder & Gräf, 2018; Juslin et al., 2003), which are arguably less sensitive to the exact value of the *s* parameter and thus less affected by the results reported in this work.

One possible solution, which we presented here in the paper, is the latent-mixture extension of the original exemplar model shown in Fig. 2. As demonstrated in the simulation, the integration of the possibility of direct recall of learned exemplars ensures a valid estimation of the parameter of interest. Furthermore, this latent-mixture model is generally preferred over the original exemplar model, when participants remembered at least some exemplars correctly. However, so far, the model assumes a very simple and error-free direct retrieval process of the criterion value of a learned exemplar in a trial, where the corresponding criterion value of the exemplar is always correctly remembered, for example, there is no confusion between similar exemplars. In addition, there might be other possible solutions, such as splitting the data into correctly remembered exemplars and other stimuli, as demonstrated in the simulation. Although this was our initial idea of fixing this issue, this approach has several disadvantages over the latent-mixture approach. For instance, the split-solution is based on the post-hoc evaluation of the observed data, where the data is divided into two different sets (recalled exemplars vs. not recalled exemplars and new stimuli) and model parameters are then estimated separately for each set. In this dichotomization procedure, it would also be an approximation to categorize all exactly remembered exemplars in the set representing the pure recall process and all other trials in the second set, and then estimating one overall *s* parameter for each set. Furthermore, the latent-mixture model models the underlying psychological processed explicitly. Another possible solution would be to either do not give participants feedback about the exact criterion value (e.g., Pachur & Olsson, 2012) or to include some exemplars in the training phase for which no feedback about the criterion value is given, similar to Experiment 2 of Bröder et al., (2017), and then estimate the *s* parameter only on these exemplars.

## Limitations

There are some limitations to this work. First, throughout this article, we focused on Medin and Schaffer’s (1978) context model extended to continuous judgment (Juslin and Persson, 2002; Juslin et al., 2003) as an exemplar model. However, several multiple-cue judgment studies (e.g., Hoffmann et al., 2014; Scheibehenne & Pachur, 2015; Pachur & Olsson, 2012) use another exemplar model, the GCM of Nosofsky (1984). We conjecture that the results found here for the *s* parameter of the context model extend to the sensitivity parameter *c* (also sometimes denoted as *h*) of the GCM as well, since the context model (Medin and Schaffer, 1978) is a special case of the generalized context model (Nosofsky, 1984) and the *s* parameter of the context model is related to the sensitivity parameter *c* through a monotonic function (see the supplemental materials). A second limitation is that for reasons of simplicity, we did not manipulate or randomize some factors in the simulation such as the general form of the criterion value function (e.g., linear, cubic, exponential), the number of cues, or the dimensionality of cues (binary vs. non-binary). However, since the biased estimation of the *s* parameter is due to having judgments of exemplars identical to the criterion value of the exemplar, irrelevant of how the criterion value is computed or how many cues there are, we expect the effects would be the same. Third, the data sets we reanalyzed originate from one lab-group and used similar materials (i.e., cue patters, criterion value function, criterion values, and exemplars). Different experimental materials may differ in the magnitude of effects we reported here. However, we expect that since the effects stem from the combination of the paradigm, the unmodified transfer of the model to this paradigm, and the estimation procedure used in multiple-cue judgment studies, and that we found the same effects in the simulation which used somewhat different materials (randomized criterion value functions, criterion values, and exemplars), the results should generalize to other studies as well.

## Conclusions

We showed that the paradigm commonly used in multiple-cue judgment research in combination with the way models are fitted to the data can lead to biased estimates and impaired validity of parameters, as well as negatively affect the fit of the models. Researchers should be aware of the different possible psychological processes underlying their data and incorporate it in their analysis or experimental design if necessary.

## Footnotes

1 There is also empirical data showing that this simplified version also outperforms the more complex model with a separate *s*_*i*_ parameter for each cue *i* in predicting individuals behavior (von Helversen & Rieskamp, 2008).

2 All simulations and analyses were conducted using R (Version 4.0.2; R Core Team, 2020b) and the R-packages *afex* (Version 0.27.2; Singmann, Bolker, Westfall, Aust, & Ben-Shachar, 2020), *doSNOW* (Version 1.0.18; Corporation & Weston, 2019), *dplyr* (Version 1.0.0; Wickham, François, Henry, & Müller, 2020), *foreach* (Version 1.5.0; Microsoft & Weston, 2020), *foreign* (Version 0.8.80; R Core Team, 2020a), *ggplot2* (Version 3.3.2; Wickham, 2016), *lsr* (Version 0.5; Navarro, 2015), *MCMCvis* (Version 0.14.0; Youngflesh, 2018), *polspline* (Version 1.1.19; Kooperberg, 2020), *psych* (Version 1.9.12.31; Revelle, 2019), *purrr* (Version 0.3.4; Henry & Wickham, 2020), *Rcpp* (Version 1.0.5; Eddelbuettel & François, 2011; Eddelbuettel & Balamuta, 2017), *reshape2* (Version 1.4.4; Wickham, 2007), *runjags* (Version 2.0.4.6; Denwood, 2016), *tibble* (Version 3.0.1; Müller & Wickham, 2020), and *truncnorm* (Version 1.0.8; Mersmann, Trautmann, Steuer, & Bornkamp, 2018). The entire article was written with the *papaja*-package (Version 0.1.0.9997; Aust & Barth, 2020)

3 We thank an anonymous Reviewer for this suggestion.

4 We used a value of .1 instead of 0, as this made it easier to ensure that there were at least two recalled exemplars, since it would lead to some problems later in the simulation when there were no or only one recalled exemplar when we estimated two separate *s* parameters on the two distinct subsets of data.

5 We used the means and standard deviations of the unbiased $\hat {s}_{int}$ estimates to define the distribution of true *s* values in this simulation.

6 These values were chosen randomly and are not based on empirical data.
